# Tri­cyclo­[3.3.1.0^3,7^]nonane-3,7-diyl bis­(4-methyl­benzene­sulfonate)

**DOI:** 10.1107/S1600536813023234

**Published:** 2013-08-23

**Authors:** Savvas Ioannou, Eleni Moushi

**Affiliations:** a Chemistry Department, University of Cyprus, Nicosia, 1678, Cyprus

## Abstract

The title compound, C_23_H_26_O_6_S_2_ was synthesized by esterification of tri­cyclo­[3.3.1.0^3,7^]nonane-3,7-diol with *p*-toluene­sulfonyl chloride. The mol­ecule has symmetry 2 and is situated on site 4*e*. The C—C bond length between the quartenary C atoms is 1.598 (2) Å, which is considerably longer than other C—C bonds in the mol­ecule. There are C—H⋯O inter­actions present in the structure. As a consequence, the packing of the molecule (viewed along [100]) appears as chains where the molecules run parallel, but each chain has the opposite direction to the neighboring ones.

## Related literature

For reviews on noradamantene and analogous pyramidalized alkenes, see: Borden (1989 ▶, 1996 ▶); Vazquez & Camps (2005 ▶). For tosyl­ates, see: Hoffman (1965 ▶). For related structures, see: Ioannou & Nicolaides (2009 ▶); Ioannou *et al.* (2010 ▶, 2012*a* ▶), and for polycyclic compounds prepared from noradamantene, see: Ioannou *et al.* (2012*b* ▶,*c* ▶, 2013 ▶). For a description of the Cambridge Crystallographic Database, see: Allen (2002 ▶).
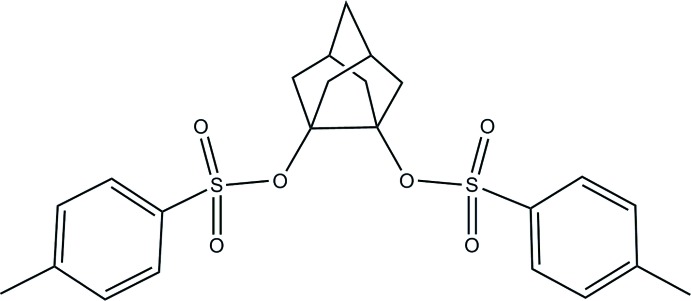


## Experimental

### 

#### Crystal data

C_23_H_26_O_6_S_2_
*M* *_r_* = 462.58
Monoclinic, 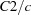

*a* = 22.3068 (8) Å
*b* = 7.5667 (2) Å
*c* = 12.7114 (5) Å
β = 98.837 (4)°
*V* = 2120.07 (13) Å^3^
*Z* = 4
Mo *K*α radiation
μ = 0.29 mm^−1^
*T* = 100 K
0.68 × 0.20 × 0.05 mm

#### Data collection

Oxford Diffraction SuperNova diffractometer
Absorption correction: multi-scan (*CrysAlis RED*; Oxford Diffraction, 2009 ▶) *T*_min_ = 0.933, *T*_max_ = 0.986
17770 measured reflections
2420 independent reflections
2210 reflections with *I* > 2σ(*I*)
*R*_int_ = 0.034

#### Refinement

*R*[*F* ^2^ > 2σ(*F* ^2^)] = 0.032
*wR*(*F* ^2^) = 0.089
*S* = 1.09
2420 reflections
142 parameters
H-atom parameters constrained
Δρ_max_ = 0.46 e Å^−3^
Δρ_min_ = −0.40 e Å^−3^

### 

Data collection: *CrysAlis CCD* (Oxford Diffraction, 2009 ▶); cell refinement: *CrysAlis CCD*; data reduction: *CrysAlis RED* (Oxford Diffraction, 2009 ▶); program(s) used to solve structure: *SHELXS97* (Sheldrick, 2008 ▶); program(s) used to refine structure: *SHELXL97* (Sheldrick, 2008 ▶); molecular graphics: *DIAMOND* (Brandenburg, 1999 ▶) and *Mercury* (Macrae *et al.*, 2006 ▶); software used to prepare material for publication: *WinGX* (Farrugia, 2012 ▶).

## Supplementary Material

Crystal structure: contains datablock(s) I. DOI: 10.1107/S1600536813023234/fb2286sup1.cif

Structure factors: contains datablock(s) I. DOI: 10.1107/S1600536813023234/fb2286Isup2.hkl

Supplementary material file. DOI: 10.1107/S1600536813023234/fb2286Isup3.cdx

Supplementary material file. DOI: 10.1107/S1600536813023234/fb2286Isup4.cml

Additional supplementary materials:  crystallographic information; 3D view; checkCIF report

## Figures and Tables

**Table 1:** Hydrogen-bond geometry (Å, °)

*D*—H⋯*A*	*D*—H	H⋯*A*	*D*⋯*A*	*D*—H⋯*A*
C4—H4*A*⋯O3^i^	0.99	2.49	3.4714 (16)	171
C10—H10⋯O2^ii^	0.95	2.57	3.2551 (19)	129
C12—H12*C*⋯O2^iii^	0.98	2.49	3.4444 (19)	164

Symmetry codes: (i) 
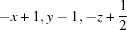
; (ii) 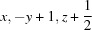; (iii) 
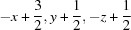
.

**Fig. 1.**
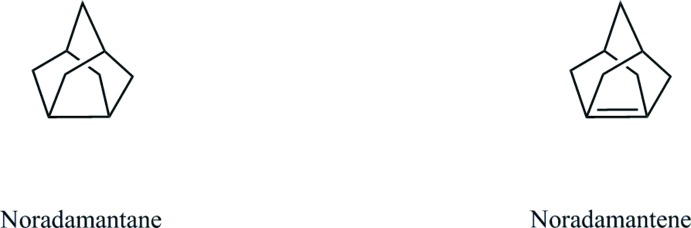
Schemes of noradamantane and noradamantene.

**Fig. 2.**
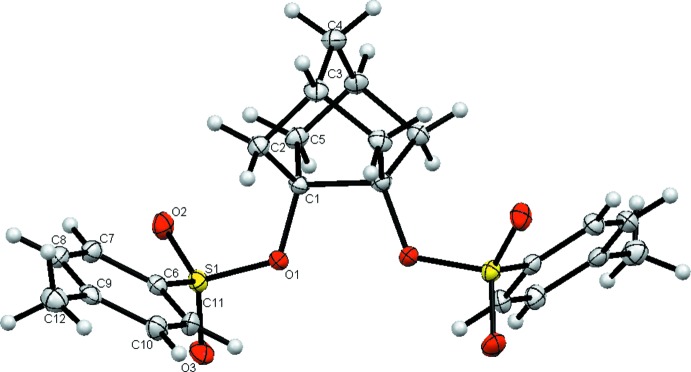
The title molecule of tricyclo-[3.3.1.03,7]nonane-3,7-diylditosylate with the atom-labelling scheme. The displacement ellipsoids are drawn at the 50% probability level.

## supplementary crystallographic information

### 1. Comment

The tosyl group is one of the best leaving groups (Hoffman, 1965). For this reason, the title compound was synthesized in attempt to form new good precursors for noradamantene (Fig. 1, Borden (1989, 1996); Vazquez & Camps, 2005). Analogous studies have already been carried out by our research group (Ioannou & Nicolaides, 2009, Ioannou *et al.*, 2010, Ioannou & Moushi, 2012*a*) on other molecules with the same noradamantane skeleton (Ioannou *et al.*, 2010, Ioannou & Moushi, 2012a investigated the same molecules which have been described in Ioannou & Nicolaides, 2009). Synthesis of noradamantene is important for the building of larger polycyclic compounds (Ioannou & Moushi (2012*b*, 2012*c*), Ioannou *et al.*, 2013).

The title compound has a 2-fold symmetry (Fig. 2). The C–C bond distance of the quaternary carbons C1— C1^i^ where (i): 1 - *x*, *y*, -*z*+1/2 was found equal to 1.598 (2) Å, which is considerably longer compared to the other C—C bonds in the title molecule. On the other hand, this long bond is comparable to those found in DUNTAI, *i.e.* tricyclo-[3.3.1.0^3,7^]nonane-3,7-diyldimesylate (Ioannou *et al.*, 2010) with the pertinent C—C bond length equal to 1.597 (3) Å, and in PAVYES, *i. e.*2,4-dioxa-λ^6^- thiatetracyclo[5.3.1.1^5,9^.0^1,5^]dodecane-3,3-dione (Ioannou & Moushi, 2012*a*) with the pertinent C—C bond length equal to 1.581 (3) Å. For the *REFCODES*, see the Cambridge Crystal Structure Database, version 5.34 (Allen, 2002).

These three compounds have the same noradamantane skeleton (Fig. 1) but different ligands at the C1 and C1^i^-positions. There are present weak C—H···O interactions in the structure (Table 1).

### 2. Experimental

4-Toluenesulfonyl chloride (1.240 g, 6.5 mmol) was added slowly at room temperature under stirring into a round bottom flask containing a solution of tricyclo-[3.3.1.0^3,7^]nonane-3,7-diol (100 mg, 0.65 mmol) in pyridine (2 ml). The mixture was refluxed at 115°C for 4 h and let to cool down to room temperature. H_2_O (20 ml) was added and the mixture was stirred for 5 min at room temperature. A white insoluble solid had formed which was separated by filtration under vacuum. The solid was dissolved in a mixture (10 ml) of hexane:dichloromethane in proportion 2:8. After slow evaporation of about a half of the solvent, colourless needle-like crystals of the title compound with typical length of 4 mm were formed (145 mg, 48% yield). M.p. 146–148°C, *δ_H_* (300 MHz, CDCl_3_), 1.44 (*s*, 2H, CH_2-bridge_), 2.18 (*d*, *J*= 7.5 Hz, 4H, CH_2(_*_a_*_)_), 2.33 (*d*, *J*= 11.1 Hz, 4H, CH_2(_*_b_*_)_), 2.38 (*s*, 2H, CH), 2.43 (*s*, 6H, CH_3_) 7.28 (*d*, *J*= 7.8 Hz, 4H, CH_Ar_), 7.79 (*d*, *J*= 7.2 Hz, 4H, CH_Ar_); *δ_C_* (75.5 MHz, CDCl_3_) 21.6 (CH_3_), 32.2 (CH_2-bridge_), 35.0 (CH), 47.4 (CH_2_), 91.2 (COTs), 127.5 (CH_Ar_), 129.3 (CH_Ar_), 135.9 (C_Ar_), 144.2 (C_Ar_).

### 3. Refinement

All the H atoms were discernible in the difference electron density map. However, they were situated into the idealized positions and refined with the following constraints: C—H = 0.95 Å, *U*_iso_(H)=1.2*U*_eq_(C) for aryl, and C—H = 0.98 Å, *U*_iso_(H)=1.5*U*_eq_(C) for the methyl atoms. The methyls were allowed to rotate about the *C*—C_methyl_ bonds using the function AFIX 137 of *SHELXL-*97 (Sheldrick, 2008). The atom H4B which is symmetry equivalent to H4A has been treated as a dummy atom with zero occupation.

### Crystal data

**Table:**

C_23_H_26_O_6_S_2_	*F*(000) = 976
*M**_r_* = 462.58	*D*_x_ = 1.449 Mg m^−^^3^
Monoclinic, *C*2/*c*	Melting point = 418–421 K
Hall symbol: -C 2yc	Mo *K*α radiation, λ = 0.71073 Å
*a* = 22.3068 (8) Å	Cell parameters from 8343 reflections
*b* = 7.5667 (2) Å	θ = 3.7–28.8°
*c* = 12.7114 (5) Å	µ = 0.29 mm^−^^1^
β = 98.837 (4)°	*T* = 100 K
*V* = 2120.07 (13) Å^3^	Needle, colourless
*Z* = 4	0.68 × 0.20 × 0.05 mm

### Data collection

**Table:**

Oxford Diffraction SuperNova diffractometer	2420 independent reflections
Radiation source: sealed X-ray tube, Dual Cu and Mo	2210 reflections with *I* > 2σ(*I*)
Mirror monochromator	*R*_int_ = 0.034
Detector resolution: 10.4223 pixels mm^-1^	θ_max_ = 27.5°, θ_min_ = 2.9°
ω scans	*h* = −28→28
Absorption correction: multi-scan (*CrysAlis RED*; Oxford Diffraction, 2009)	*k* = −9→9
*T*_min_ = 0.933, *T*_max_ = 0.986	*l* = −16→15
17770 measured reflections	

### Refinement

**Table:**

Refinement on *F*^2^	Primary atom site location: structure-invariant direct methods
Least-squares matrix: full	Secondary atom site location: difference Fourier map
*R*[*F*^2^ > 2σ(*F*^2^)] = 0.032	Hydrogen site location: difference Fourier map
*wR*(*F*^2^) = 0.089	H-atom parameters constrained
*S* = 1.09	*w* = 1/[σ^2^(*F*_o_^2^) + (0.0398*P*)^2^ + 2.9805*P*] where *P* = (*F*_o_^2^ + 2*F*_c_^2^)/3
2420 reflections	(Δ/σ)_max_ = 0.002
142 parameters	Δρ_max_ = 0.46 e Å^−^^3^
0 restraints	Δρ_min_ = −0.40 e Å^−^^3^
55 constraints	

### Special details

**Table:**

Geometry. All e.s.d.'s (except the e.s.d. in the dihedral angle between two l.s. planes) are estimated using the full covariance matrix. The cell e.s.d.'s are taken into account individually in the estimation of e.s.d.'s in distances, angles and torsion angles; correlations between e.s.d.'s in cell parameters are only used when they are defined by crystal symmetry. An approximate (isotropic) treatment of cell e.s.d.'s is used for estimating e.s.d.'s involving l.s. planes.
Refinement. Refinement of *F*^2^ against ALL reflections. The weighted *R*-factor *wR* and goodness of fit *S* are based on *F*^2^, conventional *R*-factors *R* are based on *F*, with *F* set to zero for negative *F*^2^. The threshold expression of *F*^2^ > σ(*F*^2^) is used only for calculating *R*-factors(gt) *etc*. and is not relevant to the choice of reflections for refinement. *R*-factors based on *F*^2^ are statistically about twice as large as those based on *F*, and *R*- factors based on ALL data will be even larger.

### Fractional atomic coordinates and isotropic or equivalent isotropic displacement parameters (Å^2^)

**Table:**

	*x*	*y*	*z*	*U*_iso_*/*U*_eq_	Occ. (<1)
S1	0.598720 (15)	0.45011 (4)	0.13850 (3)	0.01580 (11)	
O1	0.54196 (4)	0.40633 (13)	0.19292 (7)	0.0148 (2)	
O2	0.60807 (5)	0.31585 (15)	0.06414 (8)	0.0211 (2)	
O3	0.58926 (5)	0.62764 (14)	0.10271 (8)	0.0227 (2)	
C1	0.52716 (6)	0.22369 (17)	0.21565 (10)	0.0137 (3)	
C2	0.57714 (6)	0.11868 (19)	0.28343 (11)	0.0174 (3)	
H2A	0.6019	0.1940	0.3371	0.021*	
H2B	0.6038	0.0584	0.2393	0.021*	
C3	0.53837 (7)	−0.01293 (19)	0.33508 (12)	0.0188 (3)	
H3	0.5634	−0.0863	0.3909	0.023*	
C4	0.5000	−0.1274 (3)	0.2500	0.0212 (4)	
H4A	0.4729	−0.2043	0.2846	0.025*	
H4B	0.5271	−0.2043	0.2154	0.025*	0.0
C5	0.50275 (6)	0.11637 (19)	0.11699 (11)	0.0176 (3)	
H5A	0.5356	0.0544	0.0877	0.021*	
H5B	0.4795	0.1908	0.0609	0.021*	
C6	0.65974 (6)	0.44679 (18)	0.24360 (11)	0.0156 (3)	
C7	0.71210 (7)	0.35649 (19)	0.23109 (12)	0.0190 (3)	
H7	0.7143	0.2927	0.1674	0.023*	
C8	0.76125 (7)	0.35997 (19)	0.31229 (12)	0.0201 (3)	
H8	0.7976	0.3001	0.3033	0.024*	
C9	0.75821 (7)	0.44953 (18)	0.40641 (12)	0.0183 (3)	
C10	0.70486 (7)	0.53899 (19)	0.41720 (12)	0.0202 (3)	
H10	0.7023	0.6011	0.4813	0.024*	
C11	0.65566 (7)	0.53928 (19)	0.33660 (12)	0.0192 (3)	
H11	0.6197	0.6016	0.3446	0.023*	
C12	0.81125 (7)	0.4503 (2)	0.49473 (13)	0.0244 (3)	
H12A	0.8379	0.3501	0.4860	0.037*	
H12B	0.7966	0.4406	0.5633	0.037*	
H12C	0.8339	0.5609	0.4926	0.037*	

### Atomic displacement parameters (Å^2^)

**Table:**

	*U*^11^	*U*^22^	*U*^33^	*U*^12^	*U*^13^	*U*^23^
S1	0.01584 (18)	0.01730 (19)	0.01524 (18)	−0.00015 (12)	0.00556 (13)	0.00160 (12)
O1	0.0150 (5)	0.0138 (5)	0.0165 (5)	0.0000 (4)	0.0057 (4)	0.0016 (4)
O2	0.0217 (5)	0.0264 (6)	0.0169 (5)	−0.0014 (4)	0.0079 (4)	−0.0033 (4)
O3	0.0227 (5)	0.0210 (6)	0.0252 (6)	−0.0003 (4)	0.0065 (4)	0.0083 (4)
C1	0.0149 (6)	0.0126 (6)	0.0140 (6)	−0.0003 (5)	0.0036 (5)	0.0010 (5)
C2	0.0159 (6)	0.0174 (7)	0.0186 (7)	0.0023 (5)	0.0016 (5)	0.0025 (5)
C3	0.0199 (7)	0.0170 (7)	0.0191 (7)	0.0020 (5)	0.0024 (5)	0.0041 (5)
C4	0.0253 (10)	0.0140 (9)	0.0248 (10)	0.000	0.0059 (8)	0.000
C5	0.0208 (7)	0.0182 (7)	0.0140 (6)	0.0004 (5)	0.0031 (5)	−0.0027 (5)
C6	0.0162 (6)	0.0143 (6)	0.0172 (7)	−0.0014 (5)	0.0050 (5)	0.0013 (5)
C7	0.0210 (7)	0.0170 (7)	0.0201 (7)	0.0019 (5)	0.0065 (5)	−0.0014 (5)
C8	0.0184 (7)	0.0169 (7)	0.0257 (7)	0.0033 (5)	0.0057 (6)	0.0014 (6)
C9	0.0184 (7)	0.0153 (7)	0.0215 (7)	−0.0033 (5)	0.0035 (6)	0.0036 (5)
C10	0.0210 (7)	0.0213 (7)	0.0195 (7)	−0.0031 (5)	0.0065 (6)	−0.0031 (5)
C11	0.0174 (7)	0.0193 (7)	0.0227 (7)	0.0006 (5)	0.0084 (6)	−0.0023 (5)
C12	0.0215 (7)	0.0247 (8)	0.0260 (8)	−0.0017 (6)	0.0002 (6)	0.0025 (6)

### Geometric parameters (Å, º)

**Table:**

S1—O3	1.4236 (11)	C5—H5A	0.9900
S1—O2	1.4246 (11)	C5—H5B	0.9900
S1—O1	1.5685 (10)	C6—C7	1.383 (2)
S1—C6	1.7548 (15)	C6—C11	1.389 (2)
O1—C1	1.4600 (16)	C7—C8	1.386 (2)
C1—C5	1.5228 (18)	C7—H7	0.9500
C1—C2	1.5237 (18)	C8—C9	1.386 (2)
C1—C1^i^	1.598 (2)	C8—H8	0.9500
C2—C3	1.531 (2)	C9—C10	1.394 (2)
C2—H2A	0.9900	C9—C12	1.501 (2)
C2—H2B	0.9900	C10—C11	1.382 (2)
C3—C5^i^	1.530 (2)	C10—H10	0.9500
C3—C4	1.5389 (19)	C11—H11	0.9500
C3—H3	1.0000	C12—H12A	0.9800
C4—C3^i^	1.5389 (19)	C12—H12B	0.9800
C4—H4A	0.9900	C12—H12C	0.9800
C5—C3^i^	1.530 (2)		
			
O3—S1—O2	119.37 (7)	C1—C5—H5A	111.8
O3—S1—O1	104.49 (6)	C3^i^—C5—H5A	111.8
O2—S1—O1	110.64 (6)	C1—C5—H5B	111.8
O3—S1—C6	108.44 (7)	C3^i^—C5—H5B	111.8
O2—S1—C6	108.63 (7)	H5A—C5—H5B	109.5
O1—S1—C6	104.21 (6)	C7—C6—C11	120.91 (14)
C1—O1—S1	120.64 (8)	C7—C6—S1	119.30 (11)
O1—C1—C5	113.89 (11)	C11—C6—S1	119.76 (11)
O1—C1—C2	115.94 (11)	C6—C7—C8	119.36 (13)
C5—C1—C2	109.00 (11)	C6—C7—H7	120.3
O1—C1—C1^i^	108.76 (6)	C8—C7—H7	120.3
C5—C1—C1^i^	104.17 (11)	C9—C8—C7	120.95 (13)
C2—C1—C1^i^	103.96 (11)	C9—C8—H8	119.5
C1—C2—C3	99.75 (11)	C7—C8—H8	119.5
C1—C2—H2A	111.8	C8—C9—C10	118.56 (14)
C3—C2—H2A	111.8	C8—C9—C12	120.69 (13)
C1—C2—H2B	111.8	C10—C9—C12	120.75 (14)
C3—C2—H2B	111.8	C11—C10—C9	121.38 (14)
H2A—C2—H2B	109.5	C11—C10—H10	119.3
C5^i^—C3—C2	99.64 (11)	C9—C10—H10	119.3
C5^i^—C3—C4	109.69 (11)	C10—C11—C6	118.82 (13)
C2—C3—C4	110.76 (11)	C10—C11—H11	120.6
C5^i^—C3—H3	112.0	C6—C11—H11	120.6
C2—C3—H3	112.0	C9—C12—H12A	109.5
C4—C3—H3	112.0	C9—C12—H12B	109.5
C3—C4—C3^i^	111.49 (17)	H12A—C12—H12B	109.5
C3—C4—H4A	109.3	C9—C12—H12C	109.5
C3^i^—C4—H4A	109.3	H12A—C12—H12C	109.5
C1—C5—C3^i^	99.98 (11)	H12B—C12—H12C	109.5

Symmetry code: (i) −*x*+1, *y*, −*z*+1/2.

### Hydrogen-bond geometry (Å, º)

**Table:**

*D*—H···*A*	*D*—H	H···*A*	*D*···*A*	*D*—H···*A*
C4—H4*A*···O3^ii^	0.99	2.49	3.4714 (16)	171
C10—H10···O2^iii^	0.95	2.57	3.2551 (19)	129
C12—H12*C*···O2^iv^	0.98	2.49	3.4444 (19)	164

Symmetry codes: (ii) −*x*+1, *y*−1, −*z*+1/2; (iii) *x*, −*y*+1, *z*+1/2; (iv) −*x*+3/2, *y*+1/2, −*z*+1/2.
